# Supplementary material for: Amoeboid-like migration ensures correct horizontal cell layer formation in the developing vertebrate retina
Source: eLife. 2022 May 31;11:e76408. doi: 10.7554/eLife.76408 (PMC9208757; doi:10.7554/eLife.76408)
Supplement: Supplementary file 1. [file elife-76408-supp1.docx]

**Supplementary File 1**

**Supplementary File 1a:**

Zebrafish transgenic lines

| **Zebrafish Line** | **Structures labeled** | **Reference(s)** |
| --- | --- | --- |
| Wild-type WT-AB | Control studies | RRID:ZIRC_ZL1 |
| Wild-type WT-TL | Control studies | RRID:ZIRC_ZL86 |
| Tg(vsx-1:GFP) | Cytoplasm of BCs | Kimura et al, 2008 |
| Tg(gfap:GFP) | MGs and retinal progenitors | Bernardos and Raymond, 2006 |
| Tg(ptf1a:DsRed) | Cytoplasm of ACs and HCs | (Jusuf et al. 2012) |
| Tg(lhx-1:eGFP) | Cytoplasm of HCs and PRs | (Swanhart et al., 2010);  (Boije et al., 2015) |
| *Tg(hsp70:H2B-RFP)* | Chromatin | (Dzafic et al., 2015) |
| Tg(βactin:eGFP-lap2b) | Nuclear membrane | This Study |
| Tg(βactin:maKate2-ras) | Cell membrane | (Matejcic, Salbreux and Norden 2018) |
| Tg(*atoh7*:gap-RFP) | Membranes of RGCs and PRs | (Zolessi et al., 2006) |
| Tg(actb2:mCherry-Hsa.UTRN) | Stable F-Actin filaments | (Compagnon et al., 2014) |
| Tg(ptf1a:Gal4-VP16, UAS:gap-YFP) | Membranes of ACs and HCs | (Pisharath and Parsons, 2009)  (Williams et al., 2010) |
| *Tg(ubi: ssNcan-GFP)* | Hyaluronic acid (HA)-binding domain of Neurocan fused to GFP | Gift from Huisken laboratory^1^. Originally from Kelly Smith laboratory^2^ (Grassini et al. 2018). |
| Tg(ptf1a:Gal4-VP16, UAS:gap-YFP) | Membranes of ACs and HCs | (Pisharath and Parsons, 2009)  (Williams et al., 2010) |
| Tg(*hsp70:Lmna-mKate2*) | Nuclear membrane | This Study |
| Tg(*H2A:mCherry*) | Nuclei | (Knopf et al. 2011) |

^1.^ Jan Huisken, Morgridge Institute for Research, USA

^2.^ Kelly Smith, Institute for molecular biosciences, University of Queensland, Australia

**Supplementary File 1b:**

List of DNA constructs

| **Construct** | **Structures labeled** | **Reference** |
| --- | --- | --- |
| trβ2:tdTomato | Cytoplasm of HCs and PRs | (Suzuki et al., 2013)  Gift from the Wong laboratory^1^ |
| trβ2:membraneYFP | Membrane of HCs and PRs | (Suzuki et al., 2013)  Gift from the Wong laboratory^1^ |
| hsp70:EGFP-LAP2b | nuclear membrane | (Yanakieva et al. 2019) |
| hsp70:H2B-RFP | Chromatin | (Strzyz et al., 2015) |
| hsp70:LmnA-mKate2 | Lamin A | (Yanakieva et al. 2019) |
| T2_ βactin-actin::GFP-centrin | Centrosome | This Study |
| pCS2+ Lifeact-GFP | All F-actin | Gift from the Oates laboratory |
| pTol βactin:mKate Utrophin | Stable F-actin | This Study |

^1.^ Rachel Wong, University of Washington, USA

**Supplementary File 1c:**

List of antibodies used for immunofluorescence

| **Antibody** | **Structures labeled** | **dilution** | **RRIDs** |
| --- | --- | --- | --- |
| Anti-Laminin | Laminin α1 | 1:100 | Sigma Aldrich, L9393, RRID:AB_477163 |
| Zn-5 | RGCs | 1:50 | ZIRC, RRID:AB_10013770 |
| Alexa Fluor 647 Phalloidin | F-actin | 1:500 | Cell Signaling |
| Collagen IV | Collagen type IV | 1:100 | Abcam |
| DAPI | Chromatin | 1:1000 | Thermo Fisher Scientific |

**Supplementary File 1d:**

List of transgenes, DNA constructs, antibodies and their corresponding figures and videos

| **Zebrafish Line/ DNA construct/ antibody** | **Structures labeled** | **Figure/ Video** |
| --- | --- | --- |
| Tg(vsx-1:GFP) x Tg(ptf1a:dsRed) | Cytoplasm of BCs/ HCs, ACs | Figure 1C,  Figure 1-figure supplement 1D,  Video 3 |
| Tg(gfap:GFP) x Tg(ptf1a:dsRed) | MGs/ Cytoplasm of HCs, ACs | Figure 1E  Figure 1-figure supplement 1E-E’’ |
| Tg(lhx-1:eGFP) x Tg(ptf1a:dsRed) | Cytoplasm of HCs/ Cytoplasm of ACs and PRs | Figure 1-figure supplement 1A,  Video 1 |
| Tg(ptf1a:Gal4-VP16, UAS:gap-YFP) x Tg(*atoh7*:gap-RFP) | Membrane of HCs, ACs/ Cytoplasm of RGCs, PRs | Figure 1-figure supplement 1C,  Figure 4A-C’’,  Figure 4-figure supplement 2B-B’,  Video 2, Video 7 |
| Tg(lhx-1:eGFP) +  anti-Laminin | Cytoplasm of HCs, ACs/  Laminin α1 | Figure 2A |
| Tg(lhx-1:eGFP) x Tg(HA-GFP) | Cytoplasm of HCs, ACs/ HA | Figure 2B |
| Tg(lhx-1:eGFP) x Tg(*atoh7*:gap-RFP) | Cytoplasm of HCs, PRs/ Cytoplasm of RGCs, PRs | Figure 2D-E’’,  Figure 4-figure supplement 1D  A,B,  Figure 6, figure supplement 1A,B |
| Tg(*H2A:mCherry*) + anti-Collagen IV | Nuclei + Collagen IV | Figure 2-figure supplement 1A |
| Tg(lhx-1:eGFP) x Tg(*βactin*:maKate2-ras) | Cytoplasm of HCs, PRs/ all membranes | Figure 2-figure supplement 1B-B’ |
| Tg(*βactin*:maKate2-ras) x Tg(*βactin:lap2b-eGFP)* | All membrane/ all nuclei membranes | Figure 2-figure supplement 1C-C’ |
| hsp70:EGFP-LAP2b +  trβ2:tdTomato plasmid | nuclei membrane/  Cytoplasm of HCs, PRs | Figure 3A-A’’ |
| Tg(lhx-1:eGFP) x *Tg(hsp70:H2B-RFP)* | Cytoplasm of HCs, PRs/ nuclei | Figure 3F-F’,  Figure 3-figure supplement 2A-A’ |
| Tg(lhx-1:eGFP)  Transplanted into wild-type | Cytoplasm of HCs, PRs | Figure 3-figure supplement 1A-A’’ |
| hsp70:EGFP-LAP2b +  trβ2:tdTomato plasmids | nuclei membrane/  Cytoplasm of HCs, PRs | Figure 3-figure supplement 1E-E‘ |
| Tg(ptf1a:dsRed) + hsp70:EGFP-LAP2b plasmid | Cytoplasm of HCs, ACs/ nuclei membrane | Figure 3-figure supplement 2C-C’,  Video 5 |
| pCS2+ Lifeact-GFP +  trβ2:tdTomato plasmids | All F-Actin filaments/ Cytoplasm of HCs, PRs | Figure 4D,  Video 9 |
| pTol βactin:mKate Utrophin +  trβ2:membraneYFP plasmids | Stable F-Actin filaments/ membrane of HCs, PRs | Figure 4E-F’,  Figure 4-figure supplement 1C,  Video 10 |
| T2_βactin::GFP-centrin +  trβ2:tdTomato plasmids | centrosome/ Cytoplasm of HCs, PRs | Figure 4D-E,  Figure 4-figure supplement 2A-A’ |
| Tg(ptf1a:Gal4-VP16, UAS:gap-YFP) x *Tg(hsp70:H2B-RFP)* | Membranes of ACs and HCs/ nuclei | Figure 4-figure supplement 1A |
| Tg(lhx-1:eGFP) +  Phalloidin +DAPI | Cytoplasm of HCs, PRs/ phalloidin/ DAPI | Figure 5A |
| Tg(lhx-1:eGFP) x *Tg(hsp70:LmnA-mKate2)*  +  Phalloidin +DAPI | Cytoplasm of HCs, PRs/ overexpressed LMNA/ phalloidin/ DAPI | Figure 5B |
| Tg(lhx-1:eGFP) x *Tg(hsp70:LmnA-mKate2)* | Cytoplasm of HCs, PRs/ overexpressed LMNA | Figure 5-figure supplement 1B-C |
| hsp70:LmnA-mKate2 plasmid | overexpressed LMNA | Figure 5-figure supplement 1A |
| Tg(lhx-1:eGFP) x Tg(ptf1a:dsRed)  *+*  anti-Zn5 | Cytoplasm of HCs, PRs/ Cytoplasm of HCs, ACs/ RGCs | Figure 6A |
| Tg(lhx-1:eGFP) x Tg(ptf1a:dsRed)  *+*  Phalloidin | Cytoplasm of HCs, PRs/ phalloidin | Figure 6B |

References:

Grassini, D. R., A. K. Lagendijk, J. E. De Angelis, J. Da Silva, A. Jeanes, N. Zettler, N. I. Bower, B. M. Hogan & K. A. Smith (2018) Nppa and Nppb act redundantly during zebrafish cardiac development to confine AVC marker expression and reduce cardiac jelly volume. *Development,* 145.

Jusuf, P. R., S. Albadri, A. Paolini, P. D. Currie, F. Argenton, S. Higashijima, W. A. Harris & L. Poggi (2012) Biasing amacrine subtypes in the Atoh7 lineage through expression of Barhl2. *J Neurosci,* 32**,** 13929-44.

Knopf, F., C. Hammond, A. Chekuru, T. Kurth, S. Hans, C. W. Weber, G. Mahatma, S. Fisher, M. Brand, S. Schulte-Merker & G. Weidinger (2011) Bone regenerates via dedifferentiation of osteoblasts in the zebrafish fin. *Dev Cell,* 20**,** 713-24.

Matejcic, M., G. Salbreux & C. Norden (2018) A non-cell-autonomous actin redistribution enables isotropic retinal growth. *PLoS Biol,* 16**,** e2006018.

Yanakieva, I., A. Erzberger, M. Matejcic, C. D. Modes & C. Norden (2019) Cell and tissue morphology determine actin-dependent nuclear migration mechanisms in neuroepithelia. *J Cell Biol,* 218**,** 3272-3289.
